# Direct comparison of the acute effects of lysergic acid diethylamide and psilocybin in a double-blind placebo-controlled study in healthy subjects

**DOI:** 10.1038/s41386-022-01297-2

**Published:** 2022-02-25

**Authors:** Friederike Holze, Laura Ley, Felix Müller, Anna M. Becker, Isabelle Straumann, Patrick Vizeli, Sebastian Silva Kuehne, Marc A. Roder, Urs Duthaler, Karolina E. Kolaczynska, Nimmy Varghese, Anne Eckert, Matthias E. Liechti

**Affiliations:** 1 Clinical Pharmacology and Toxicology, Department of Biomedicine and Department of Clinical Research, University Hospital Basel, Basel, Switzerland; 2 Department of Pharmaceutical Sciences, University of Basel, Basel, Switzerland; 3 Psychiatric University Hospital, University of Basel, Basel, Switzerland; 4 Transfaculty Research Platform Molecular and Cognitive Neuroscience, University of Basel, Basel, Switzerland

**Keywords:** Drug development, Human behaviour

## Abstract

Growing interest has been seen in using lysergic acid diethylamide (LSD) and psilocybin in psychiatric research and therapy. However, no modern studies have evaluated differences in subjective and autonomic effects of LSD and psilocybin or their similarities and dose equivalence. We used a double-blind, randomized, placebo-controlled, crossover design in 28 healthy subjects (14 women, 14 men) who underwent five 25 h sessions and received placebo, LSD (100 and 200 µg), and psilocybin (15 and 30 mg). Test days were separated by at least 10 days. Outcome measures included self-rating scales for subjective effects, autonomic effects, adverse effects, effect durations, plasma levels of brain-derived neurotrophic factor (BDNF), prolactin, cortisol, and oxytocin, and pharmacokinetics. The doses of 100 and 200 µg LSD and 30 mg psilocybin produced comparable subjective effects. The 15 mg psilocybin dose produced clearly weaker subjective effects compared with both doses of LSD and 30 mg psilocybin. The 200 µg dose of LSD induced higher ratings of ego-dissolution, impairments in control and cognition, and anxiety than the 100 µg dose. The 200 µg dose of LSD increased only ratings of ineffability significantly more than 30 mg psilocybin. LSD at both doses had clearly longer effect durations than psilocybin. Psilocybin increased blood pressure more than LSD, whereas LSD increased heart rate more than psilocybin. However, both LSD and psilocybin showed comparable cardiostimulant properties, assessed by the rate-pressure product. Both LSD and psilocybin had dose-proportional pharmacokinetics and first-order elimination. Both doses of LSD and the high dose of psilocybin produced qualitatively and quantitatively very similar subjective effects, indicating that alterations of mind that are induced by LSD and psilocybin do not differ beyond the effect duration. Any differences between LSD and psilocybin are dose-dependent rather than substance-dependent. However, LSD and psilocybin differentially increased heart rate and blood pressure. These results may assist with dose finding for future psychedelic research.

**Trial registration:** ClinicalTrials.gov identifier: NCT03604744

## Introduction

Lysergic acid diethylamide (LSD) and psilocybin are both classic serotonergic psychedelics that are used recreationally [1] and have recently become promising candidates for the treatment of various psychiatric disorders (e.g., anxiety disorders and major depressive disorder) and neurologic disorders (e.g., cluster headache and migraine) [2–6]. Both substances induce complex alterations of mind via stimulation of the serotonin 5-hydroxytryptamine-2A (5-HT_2A_) receptor [7–9]. LSD exerts additional activity at dopamine D_1-3_ receptors, whereas psilocin, the active metabolite of psilocybin, inhibits the serotonin transporter [10]. Whether these differences in receptor binding profiles produce differential subjective effects in humans has not yet been studied. Recent research has investigated either psilocybin or LSD alone [7, 11–15]. Differences between the two substances with regard to their acute effects, similarities, and dose-equivalence remain unclear.

Therefore, the present study evaluated and directly compared the acute subjective, autonomic, and endocrine effects of LSD and psilocybin using two doses of each substance and placebo within the same subjects. The acute subjective effects of LSD and psilocybin were determined using validated psychometric instruments that are used internationally in healthy subjects as well as in therapeutic studies in patients [3, 16–18]. In addition, we determined plasma LSD and psilocin concentrations over time and over 24 h to provide valid pharmacokinetic parameters for all substances and doses used. Previous research has shown that plasma concentrations of LSD and psilocybin are strongly linked to subjective effects [7, 19, 20]. Pharmacokinetic data are thus important and needed for the potential development of these substances into medications. Both LSD and psilocybin have previously been shown to induce dose-dependent cardiovascular stimulation and influence endocrine functions [11, 15, 21–23]. However, potential differences between these two substances are unexplored. Therefore, we assessed blood pressure, heart rate, body temperature, and endocrine effects, including plasma levels of cortisol, prolactin (PRL), and oxytocin. In addition, we assessed plasma levels of brain-derived neurotrophic factor (BDNF), which is considered a possible marker of neurogenesis [24] and has been shown to increase following psychedelic administration [7, 25–27].

## Methods and materials

### Study design

The study used a double-blind, placebo-controlled, crossover design with five experimental test sessions to investigate responses to (*i*) placebo, (*ii*) 100 µg LSD, (*iii*) 200 µg LSD, (*iv*) 15 mg psilocybin, and (*v*) 30 mg psilocybin. The order of administration was random and counterbalanced. The washout periods between sessions were at least 10 days. The study was conducted in accordance with the Declaration of Helsinki and International Conference on Harmonization Guidelines in Good Clinical Practice and approved by the Ethics Committee of Northwest Switzerland (EKNZ) and Swiss Federal Office for Public Health. The study was registered at ClinicalTrials.gov (NCT03604744).

### Participants

Twenty-eight healthy participants (14 men and 14 women; mean age ± SD: 35 ± 9.4 years; range: 25–52 years) were recruited by word of mouth or from a pool of volunteers who had contacted our research group because they were interested in participating in a clinical trial that investigated psychedelics. All of the subjects provided written informed consent and were paid for their participation. Exclusion criteria were age <25 years or >65 years, pregnancy (urine pregnancy test at screening and before each test session), personal or family (first-degree relative) history of major psychiatric disorders (assessed by the Semi-structured Clinical Interview for *Diagnostic and Statistical Manual of Mental Disorders*, 4th edition, Axis I disorders by a trained psychiatrist), the use of medications that may interfere with the study medications (e.g., antidepressants, antipsychotics, and sedatives), chronic or acute physical illness (e.g., abnormal physical exam, electrocardiogram, or hematological and chemical blood analyses), tobacco smoking (>10 cigarettes/day), lifetime prevalence of illicit drug use >10 times (except for Δ^9^-tetrahydrocannabinol), illicit drug use within the last 2 months, and illicit drug use during the study period (determined by urine drug tests). The participants were asked to consume no more than 10 standard alcoholic drinks/week and have no more than one drink on the day before the test sessions. Fourteen participants had previously used a psychedelic, including LSD (11 participants, 1–5 times), psilocybin (six participants, 1–3 times), and *O*-methyl-bufotenin (5-MeO-DMT; one participant, two times), 12 participants had used methylenedioxymethamphetamine (MDMA; 1–5 times), 13 participants had previously used a stimulant, including methylphenidate (two participants, once), amphetamine (nine participants, 1–5 times), and cocaine (six participants, 1–2 times), one participant had used 4-bromo-2,5-dimethoxyphenethylamine (2C-B; once), and one participant had used ketamine (once). Ten participants had never used any illicit drugs with the exception of cannabis.

### Study drugs

LSD base (>99% purity; Lipomed AG, Arlesheim, Switzerland) was administered as an oral solution that was produced according to good manufacturing practice in units that contained 100 µg LSD in 1 ml of 96% ethanol [19]. The exact analytically confirmed LSD base content (mean ± SD) was 84.5 ± 0.98 µg (*n* = 10 samples). Stability of the formulation for longer than the study period was documented in an identically produced previous batch [19]. Placebo consisted of identical units that were filled with ethanol only. Psilocybin (99.7% purity; ReseaChem GmbH, Burgdorf, Switzerland) was administered in opaque capsules that contained a 5 mg dose of psilocybin dihydrate and an exact analytically confirmed actual psilocybin content of 4.61 ± 0.09 mg (mean ± SD, *n* = 10 samples). The stability of both LSD and psilocybin products was confirmed after the study ended. Placebo consisted of identical opaque capsules that were filled with mannitol. A double-dummy method was used. The subjects received six capsules and two solutions in each session: (*i*) six placebo capsules and placebo/placebo solutions, (*ii*) six placebo capsules and 100 µg LSD/placebo solutions, (*iii*) six placebo capsules and 100 µg LSD/100 µg LSD solutions, (*iv*) three 5 mg psilocybin and three placebo capsules and placebo/placebo solutions, and (*v*) six 5 mg psilocybin capsules and placebo/placebo solutions. At the end of each session and at the end of the study, the participants were asked to retrospectively guess their treatment assignment.

### Study procedures

The study included a screening visit, five 25 h test sessions, and an end-of-study visit. Test days were separated by at least 10 days. The sessions were conducted in a calm hospital room. Only one research subject and one investigator were present during each test session. The test sessions began at 8:00 AM. A urine sample was taken to verify abstinence from drugs of abuse, and a urine pregnancy test was performed in women. The subjects then underwent baseline measurements. LSD, psilocybin, or placebo was administered at 9:00 AM. The outcome measures were repeatedly assessed for 24 h. Standardized lunches and dinners were served at ~1:30 PM and 6:00 PM, respectively. The subjects were never alone during the acute effect phase, and the investigator was in a room next to the subject for up to 24 h. The subjects were sent home the next day at ~9:15 AM.

### Subjective drug effects and effect durations

Subjective effects were assessed repeatedly using visual analog scales (VASs) [21, 28] 1 h before and 0, 0.25, 0.5, 0.75, 1, 1.5, 2, 2.5, 3, 3.5, 4, 5, 6, 7, 8, 9, 10, 11, 12, 14, 16, and 24 h after drug administration. The Adjective Mood Rating Scale (AMRS) [29] was used 1 h before and 3, 6, 9, 12, and 24 h after drug administration. The 5 Dimensions of Altered States of Consciousness (5D-ASC) scale [30, 31] was used as the primary outcome measure and was administered 24 h after drug administration to retrospectively rate peak drug effects. Mystical experiences were assessed 24 h after drug administration using the States of Consciousness Questionnaire [32, 33] that includes the 43-item Mystical Effects Questionnaire (MEQ43) [32], 30-item Mystical Effects Questionnaire (MEQ30) [34], and subscales for “aesthetic experience” and negative “nadir” effects. Subjective effect measurements are described in detail in the Supplementary Methods online.

The time to onset, time to maximal effect, time to offset, and effect duration were assessed using the classic pharmacokinetic-pharmacodynamic (PK-PD) link module in Phoenix WinNonlin 8.3 (Certara, Princeton, NJ, USA) using the “any drug effect” VAS effect-time plots and an onset/offset threshold of 10% of the maximum individual response as described previously in detail [7, 19].

### Autonomic and adverse effects

Blood pressure, heart rate, and tympanic body temperature were repeatedly measured at baseline and 0, 0.25, 0.5, 0.75, 1, 1.5, 2, 2.5, 3, 3.5, 4, 5, 6, 7, 8, 9, 10, 11, 12, 14, 16, and 24 h after drug administration [35]. Pupil size was assessed at baseline and 1, 2.5, 4, 7, 11, and 24 h after drug administration [21]. Adverse effects were assessed 1 h before and 12 and 24 h after drug administration using the List of Complaints (LC) [36].

### Endocrine effects and BDNF

Plasma concentrations of cortisol, PRL, oxytocin, and BDNF were determined as previously described [7, 21, 22, 28]. Cortisol, PRL, and oxytocin were measured before and 2.5 h after drug administration. Plasma BDNF levels were measured at baseline and 4, 6, and 12 h after drug administration.

### Plasma LSD and psilocin concentrations

Blood was collected into lithium heparin tubes. The blood samples were immediately centrifuged, and the plasma was subsequently stored at −80 °C until analysis. Plasma concentrations of LSD were determined by ultra-high-performance liquid chromatography tandem mass spectrometry with a lower limit of quantification of 10 pg/ml [19]. Plasma psilocin concentrations were analyzed using ultra-high-performance liquid chromatography tandem mass spectrometry as described previously [37]. Both methods were fully validated.

### Pharmacokinetic analyses

Pharmacokinetic parameters were estimated using non-compartmental methods as described previously [19]. Analyses were conducted using Phoenix WinNonlin 8.3 (Certara, Princeton, NJ, USA).

### Data analysis

Peak (E_max_ and/or E_min_) or peak change from baseline (ΔE_max_) values were determined for repeated measures. The values were then analyzed using repeated-measures analysis of variance (ANOVA), with drug as the within-subjects factor, followed by the Tukey post hoc tests using Statistica 12 software (StatSoft, Tulsa, OK, USA). The criterion for significance was *p* < 0.05.

## Results

### Subjective drug effects

**Fig. 1:**
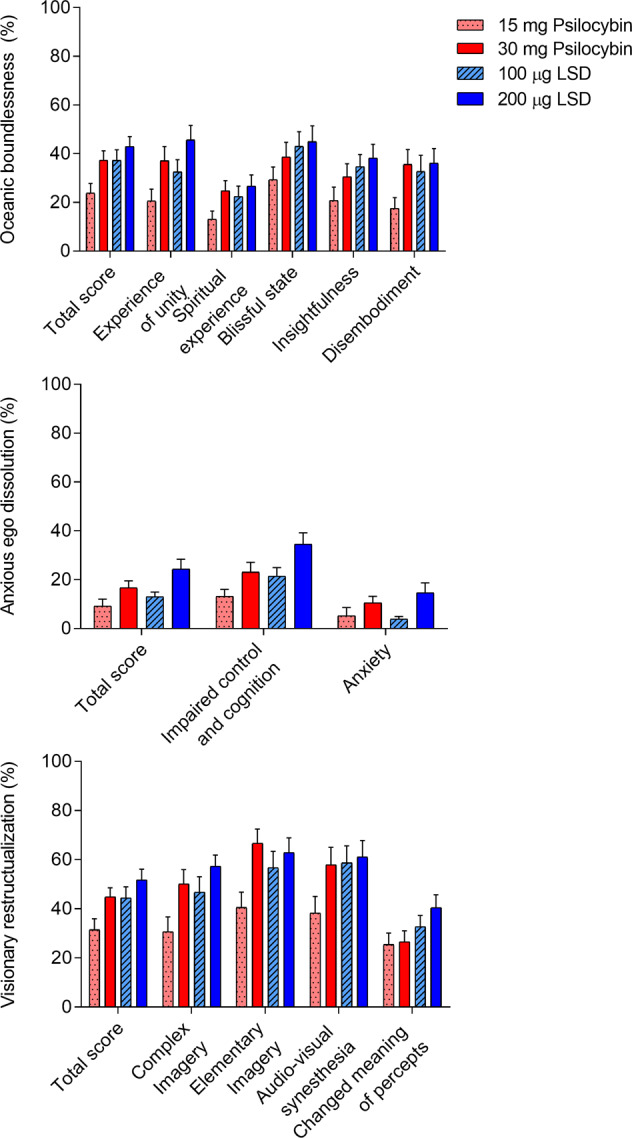
Acute alterations of mind on the 5 Dimensions of Altered States of Consciousness (5D-ASC) scale. Psilocybin at 30 mg produced alterations of mind that were nominally similar to 100 µg LSD and not significantly different from either 100 or 200 µg LSD. LSD at 100 and 200 µg significantly differed only in the “Anxious Ego Dissolution” total score and the “impaired control and cognition” and “anxiety” subscales. Effects of the 15 mg psilocybin dose were clearly lower than 100 and 200 µg LSD and 30 mg psilocybin on most subscales. Placebo scores were too low for visualization. The data are expressed as the mean ± SEM percentage of maximally possible scale scores in 28 subjects. Statistics are shown in Supplementary Table S1.

**Fig. 2:**
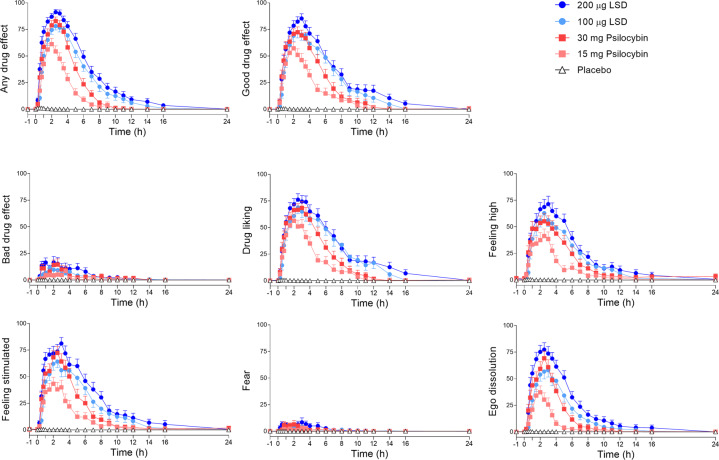
Acute subjective effects induced by lysergic acid diethylamide (LSD) and psilocybin over time on the Visual Analog Scale (VAS). LSD (100 or 200 µg), psilocybin (15 or 30 mg), or placebo was administered at *t* = 0 h. Generally, the LSD doses of 100 µg and 200 µg and psilocybin dose of 30 mg produced comparable subjective effects on the VASs “any drug effect,” “good drug effect,” “bad drug effect,” “drug liking,” “feeling high,” “feeling stimulated,” and “fear.” Only the VAS “ego dissolution” showed a significant difference between 100 and 200 µg LSD. The high 30 mg psilocybin dose produced maximal subjective effects that were comparable to 100 and 200 µg LSD, with no significant differences on any of the VASs. The 30 mg psilocybin dose produced significantly greater peak responses than the 15 mg psilocybin dose on the VAS “any drug effect,” “good drug effect,” “feeling stimulated,” and “ego dissolution.” The data are expressed as the mean ± SEM percentage of maximally possible scale scores in 28 subjects. The corresponding maximal responses and statistics are shown in Supplementary Table S3.

Alterations of mind and mystical-type effects are shown in Fig. 1 and Supplementary Fig. S1, respectively. Statistics are summarized in Supplementary Tables S1–2. Subjective effects over time on the VAS are shown in Fig. 2 and Supplementary Fig. S2. Effects on mood over time on the AMRS are shown in Supplementary Fig. S3. The corresponding peak responses and statistics are presented in Supplementary Tables S3–4. Characteristics of subjective responses derived from the PK-PD model are shown in Table 1.

**Table 1:** Characteristics of the subjective response to different doses of LSD and psilocybin.

Effect	15 mg Psilocybin	30 mg Psilocybin	100 µg LSD	200 µg LSD	*F*_3,81_	*P = *
Time to onset (h)	0.8 ± 0.3 (0.3–1.5)	0.8 ± 0.4 (0.1–1.8)	0.6 ± 0.2^#^ (0.3–1.0)	0.4 ± 0.2^***, ###^ (0.2–0.8)	12.5	<0.001
Time to offset (h)	6.4 ± 2.1 (3.7–11)	7.3 ± 2.3 (4.2–12)	10 ± 3.1^***, ###^ (6.3–8.8)	11 ± 3.6^***, ###^ (5.8–20)	31.2	<0.001
Time to maximal effect (h)	2.1 ± 0.5 (1.5–3.4)	2.3 ± 0.8 (0.5–3.3)	2.5 ± 0.5** (1.8–2.4)	2.2 ± 0.6 (1.2–3.9)	3.8	<0.05
Effect duration (h)	5.6 ± 2.2 (2.5–10)	6.5 ± 2.4 (3.7–12)	9.3 ± 3.2^***, ###^ (5.7–17)	11 ± 3.7^***, ###^ (5.4–20)	34.4	<0.001
Maximal effect (%)	58 ± 25 (13–98)	80 ± 18 *** (43–100)	77 ± 18*** (33–100)	87 ± 13*** (46–100)	20.0	<0.001

Parameters are for “any drug effects” as predicted by the PK-PD link model. The threshold to determine times to onset and offset was set individually at 10% of the individual maximal response. Values are mean ± SD (range).

^**^P  <  0.01, ^***^P < 0.001 compared with 15  mg of psilocybin; ^#^*P*  <  0.05, ^###^*P* < 0.001 compared with 30  mg of psilocybin.

LSD at doses of 100 and 200 µg and the psilocybin dose of 30 mg produced comparable subjective effects. Specifically, 200 µg LSD produced comparable positive drug effects to 100 µg LSD, but the higher dose produced greater ego-dissolution (*p* < 0.05) and a trend toward an increase in anxiety (*p* = 0.054). The high 30 mg psilocybin dose produced comparable maximal subjective effects to the 100 and 200 µg LSD doses, with no significant differences in any of the VASs or 5D-ASC subscales. The 200 µg LSD and 30 mg psilocybin doses differed only in ratings of “ineffability” on the MEQ. Effects of the 15 mg psilocybin dose were significantly lower than the 200 µg LSD dose on the VASs “any drug effect” (*p* < 0.001), “good drug effect” (*p* < 0.001), “stimulated” (*p* < 0.001), “talkative” (*p* < 0.01), “perception of time” (*p* < 0.001), and “ego-dissolution” (*p* < 0.001) and 5D-ASC total scores (*p* < 0.001) and all main dimensions, including anxious ego dissolution (*p* < 0.001), oceanic boundlessness (*p* < 0.001), and visionary restructuralization (*p* < 0.001). The 15 mg psilocybin dose produced clearly lower effects than the 30 mg psilocybin and both LSD doses.

On the AMRS, effects of 100 and 200 µg LSD were generally nominally higher than 15 and 30 mg psilocybin, and both LSD doses significantly increased “emotional excitation” compared with psilocybin.

Both LSD doses had significantly longer effect durations and an earlier onset of effects compared with both doses of psilocybin (Table 1).

There was no difference in the subjective effects of LSD and psilocybin between male and female participants.

### Autonomic and adverse effects

**Fig. 3:**
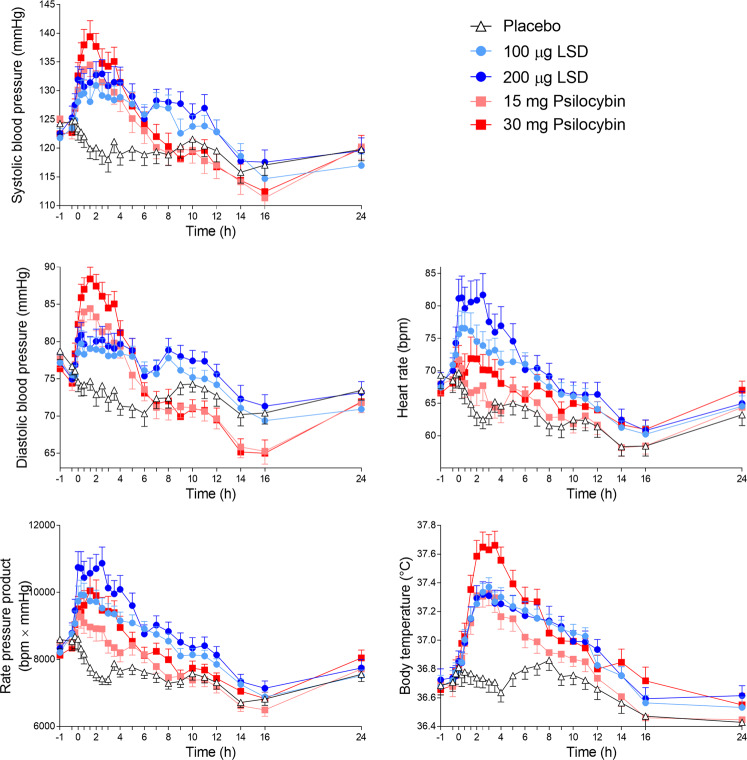
Acute autonomic effects of lysergic acid diethylamide (LSD) and psilocybin over time. The 100 and 200 µg doses of lysergic acid diethylamide (LSD) only moderately increased blood pressure compared with placebo, whereas 15 and 30 mg psilocybin induced more pronounced increases in blood pressure. The 100 and 200 µg doses of LSD markedly increased heart rate, whereas only the higher 30 mg dose of psilocybin induced a moderate increase in heart rate compared with placebo. Both the 100 and 200 μg LSD doses and the 15 mg psilocybin dose increased body temperature moderately and similarly, whereas 30 mg psilocybin induced a more pronounced increase in body temperature compared with all other conditions. LSD (100 or 200 µg), psilocybin (15 or 30 mg), or placebo was administered at *t* = 0 h. The data are expressed as the mean ± SEM in 28 subjects. Maximal effects and statistics are shown in Supplementary Table S5.

Autonomic effects over time and respective peak effects are shown in Fig. 3 and Supplementary Table S5, respectively. Frequently reported adverse effects, systematically assessed by the LC, are presented in Supplementary Table S6. Both LSD and psilocybin significantly increased diastolic and systolic blood pressure, body temperature, and pupil size compared with placebo. LSD increased blood pressure and body temperature only moderately, whereas 30 mg psilocybin produced significantly greater increases in blood pressure and body temperature compared with LSD and 15 mg psilocybin. In contrast, both LSD doses produced a greater increase in heart rate compared with both psilocybin doses and placebo. Psilocybin at a dose of 30 mg but not 15 mg moderately increased heart rate compared with placebo.

Both LSD and psilocybin increased pupil size at both doses (Supplementary Fig. S4 and Supplementary Table S5). Furthermore, 200 µg LSD and 30 mg psilocybin significantly impaired normal light-induced pupil constriction compared with placebo.

LSD and psilocybin at both doses increased the total acute (0–12 h) adverse effect score on the LC compared with placebo. Subacute (12–24 h) adverse effect scores were significantly increased by the high doses (200 µg LSD and 30 mg psilocybin) compared with placebo. There was no difference in the autonomic and adverse effects of LSD and psilocybin between male and female participants.

LSD and psilocybin also resulted in additional adverse events in the evening on the treatment day or within 48 h after treatment. Adverse aftereffects included headaches (four subjects after psilocybin and three after LSD), migraine attack (one subject after LSD), nosebleeds (one subject after psilocybin), low mood (two subjects after psilocybin and two subjects after LSD), nausea (two subjects after psilocybin and one subject after LSD), nightmares (one subject after psilocybin), restlessness (one subject after psilocybin and one subject after LSD), vivid dreams (one subject after LSD), insomnia (one subject after psilocybin), and involuntary movement of the lower extremities (one subject after LSD). Nine flashback episodes occurred in five subjects (one to 20 times within 72 h after substance administration; five episodes after LSD administration and four episodes after psilocybin administration). Altogether, the type and number of adverse events after acute psilocybin and LSD administration were comparable. No severe adverse events were observed.

### Endocrine effects and BDNF

Effects of LSD and psilocybin on plasma levels of cortisol, PRL, oxytocin, and BDNF are shown in Supplementary Fig. S5 and Supplementary Table S5. Both LSD and psilocybin significantly increased plasma cortisol, PRL, and oxytocin levels. Neither LSD nor psilocybin significantly elevated plasma BDNF levels.

### Plasma drug concentrations

The concentration-time curves for LSD and psilocin are shown in Supplementary Fig. S6. Supplementary Table S7 shows the pharmacokinetic parameters. The geometric mean maximum (C_max_) values (range) for 100 and 200 µg LSD were 1.9 (1.1–3.3) ng/ml and 3.4 (1.6–5.8) ng/ml, respectively. The T_max_ values were 1.6 (1.75–3.0) h and 1.6 (1.3–3.5) h, respectively. Elimination half-lives (t_1/2_) were 4.3 (2.5–6.0) h and 4.0 (2.3–7.1) h, respectively. The C_max_ values for 15 and 30 mg psilocin were 13 (7–21) ng/ml and 25 (14–41) ng/ml, respectively. The corresponding T_max_ values were 2.3 (0.8–3.5) h and 2.5 (0.77–4.0) h, respectively. Elimination half-lives (t_1/2_) were 2.4 (1.7–3.1) h and 2.7 (1.9–3.4) h, respectively. Both LSD and psilocybin showed linear pharmacokinetics. Body weight had no influence on the pharmacokinetics of LSD or psilocybin.

### Blinding

Data on the participants’ retrospective identification of the substance and dose are shown in Supplementary Table S8. Overall, no clear distinction between LSD and psilocybin could be made after the sessions, nor at the end of the study. The 15 mg dose of psilocybin was correctly identified in 64% of the sessions after the session and mistaken for 100 µg LSD in 29% of the sessions. When asked after the session, the 30 mg psilocybin dose was correctly identified in 57% of the sessions and mistaken for 15 mg psilocybin in 29% of the sessions. The 100 µg LSD dose was correctly identified in 57% of the sessions and mistaken for 200 µg LSD in 21% of the sessions. The 200 µg LSD dose was correctly identified in 61% of the sessions and mistaken as 100 µg LSD and 30 mg psilocybin in 18% of the sessions for both. When asked at the end of the study, 15 mg psilocybin was mostly mistaken for 30 mg psilocybin, 30 mg psilocybin was mostly mistaken for 100 µg LSD, 100 µg LSD was mostly mistaken for 15 mg psilocybin, and 200 µg LSD was mostly mistaken for 100 µg LSD. Placebo could be distinguished well from active substance and correctly identified in 96% of the sessions.

## Discussion

The present study investigated and directly compared acute effects of LSD and psilocybin using well-defined doses in healthy participants. Previous recent studies only investigated either LSD or psilocybin alone. The present study was the first modern study that compared both substances within the same study using a within-subjects design. We used LSD and psilocybin doses that covered the range of therapeutically used doses and were expected to induce comparable subjective effects as previously reported in similar Phase 1 studies of either LSD or psilocybin [11, 21, 33]. The present study was also the first to describe acute effects and particularly the pharmacokinetics of fixed doses of psilocybin, thus complementing our recent study using a fixed dose of 25 mg psilocybin in healthy subjects [38]. In contrast, several previous studies of psilocybin in healthy participants used body weight-adjusted dosing approaches [11, 13–15, 39, 40]. Fixed dosing is likely to be used in therapeutic settings [2, 41]. The body weight adjustment of the psilocybin dose did not alter subjective effects and had no advantages [42], and fixed dosing is more practical in large trials in patients. Both LSD and psilocybin were used in pharmaceutical formulations with defined content uniformity and stability. Furthermore, plasma concentrations of both LSD and psilocin were determined as measures of exposure to the substances.

Subjective effects that were induced by both doses of LSD and the high 30 mg dose of psilocybin were largely comparable, whereas 15 mg psilocybin exerted clearly weaker effects. Subjective effects of LSD in the present study were similar to previous studies that investigated either LSD or psilocybin [7, 11, 28]. For LSD, the dose-effect relationship reached a ceiling effect for good drug effects at a dose of 100 µg. Only ego dissolution and negative drug effects further increased at a dose of 200 µg compared with 100 µg, which is consistent with previous studies [7, 43]. Notably, the ceiling effect was less pronounced in the present study compared with our previous study [7]. In contrast, psilocybin showed a clear dose-effect relationship on most outcome measures at the doses used in the present study. Ratings of the high 30 mg psilocybin dose were nominally between the 100 and 200 µg doses of LSD, indicating that 30 mg psilocybin corresponds to 150 µg LSD base, a dose that was not tested herein. This means that the doses of psilocybin that were used in the present study (30 and 15 mg) were lower in terms of strength compared with the two doses of LSD, and ceiling effects were likely not reached for psilocybin compared with LSD. A previous study that investigated body weight-adjusted doses of psilocybin of ~20 mg/70 kg, 30 mg/70 kg, and 40 mg/70 kg found no difference in positive mood scale ratings on the MEQ30 [14], indicating that a ceiling effect for good drug effects for psilocybin, similar to LSD, might be reached at doses above 20 mg. The only subscale on which 200 µg LSD was significantly different from 30 mg psilocybin was “ineffability” on the MEQ30 and MEQ43. Ineffability largely describes the ability to express an experience in words. Because 200 µg LSD was nominally more effective than 30 mg psilocybin and because 100 µg LSD showed a nearly significant lower effect on this scale compared with 200 µg LSD, we suggest that this is a dose effect rather than a substance-specific effect. In addition, no sex differences were found, consistent with previous reports [7, 28, 43].

Both LSD and psilocybin had dose-dependent effect durations, with higher doses producing longer effects. However, the effects of LSD were also clearly and significantly longer than the effects of psilocybin. The differences in the duration of action can be fully explained by differences in the pharmacokinetics of LSD and psilocin. The elimination half-life values of LSD and psilocin were an average of ~4 h and 2.5 h, respectively. These values are consistent with previous studies [7, 13, 19, 37], although a slightly shorter half-life of 2 h has also been described for psilocybin [38, 44]. Body weight had no influence on LSD or psilocin plasma concentrations, as described previously [7, 38, 45]. The faster time of onset for LSD can be explained by the liquid formulation compared with the capsules that were used for psilocybin and cannot be attributed to the substance.

LSD and psilocybin both produced significant autonomic stimulant effects as observed previously [7, 11, 40, 43]. The cardiostimulant responses were present at both doses, with a trend toward greater responses at the higher doses. Interestingly, psilocybin produced stronger elevations of arterial blood pressure, whereas LSD produced stronger elevations of heart rate. When combining elevations of heart rate and blood pressure into the rate-pressure product, the high dose of psilocybin (30 mg) and both doses of LSD (100 and 200 µg) exerted overall similar cardiovascular stimulation, whereas the 15 mg dose of psilocybin exerted overall weaker effects. Psilocybin increased body temperature more than LSD. Psilocybin also produced greater impairments in pupil contraction compared with LSD. This lower pupillary contraction in response to a light stimulus has also been observed with MDMA compared with LSD [28] and may represent a similarity of psilocybin and MDMA. This similarity may be explained by a common action of psilocybin and MDMA on the serotonin transporter [10]. Overall, however, these autonomic effects were moderate and transient and thus not a safety concern. We also assessed acute and subacute adverse effects and spontaneously reported adverse events between test days. LSD and psilocybin produced comparable acute adverse effects, but the high doses (30 mg psilocybin and 200 µg LSD) produced more subacute adverse effects, indicating that higher doses are associated with longer-lasting and more unpleasant effects. The number and type of systematically assessed and spontaneous reported adverse effects are comparable to those reported in a larger pooled analysis of the safety of LSD in healthy subjects [43].

The true content of the LSD formulation in the present study was 11–14% lower than in previous studies by our group that used the same dose and formulation [7, 19, 28]. This lower LSD content and larger study group size might partly explain the trend toward greater effect differences between the 100 and 200 µg LSD doses compared with a previous dose-finding study in 16 subjects [7]. The two doses of psilocybin (15 and 30 mg) that were selected for this study were lower in terms of acute effect strength compared with the 100 and 200 µg doses of LSD. We also recently evaluated the acute effects of 25 mg psilocybin. Altogether, these results suggest that 20 mg psilocybin is equivalent to 100 µg LSD, and 30 mg psilocybin is equivalent to 150 µg LSD, a consistency that was also noted elsewhere [46]. Thus, the dose equivalence of LSD to psilocybin is ~1:200. This result may be helpful for dose finding in future studies and facilitate interpretations of future clinical results that are obtained with either substance.

The present study was well blinded. The only condition that was identified by the subjects with high certainty was placebo. Furthermore, the high dose of LSD (200 µg) was almost never mistaken for the low dose of psilocybin (15 mg). Generally, both the low and high doses were more likely to be confused with each other rather than the high dose being exclusively mistaken for the low dose. Interestingly, this was still the case at the end of the study, despite the clear differences in effect durations between LSD and psilocybin that could be expected to unmask the blinding between substances. These findings further support the view that alterations of states of consciousness that are induced by LSD and psilocybin are more likely dose-dependent rather than substance-dependent and that the differences in their pharmacological profiles [10] do not relevantly influence subjectively experienced effects. Studies in rats indicated a later, more negative, temporal phase with involvement of D_2_ and D_4_ receptors for LSD, but not for psilocybin [47, 48]. The finding of no difference in the quality of subjective effects of LSD and psilocybin also confirms that both classic psychedelics produce their effects via shared agonistic effects on 5-HT_2A_ receptors. The subjective effects of both substances can robustly be blocked by 5-HT_2A_ receptor antagonist administration in humans [7–9].

In the present study, neither LSD nor psilocybin altered plasma BDNF concentrations compared with placebo. Previous studies reported that 200 µg LSD but not 100 µg LSD increased BDNF levels [7, 28]. In addition, LSD and psilocybin both increased PRL and cortisol levels, which are markers of serotonergic activity [49]. Furthermore, the present study was the first to document increases in circulating oxytocin after psilocybin administration as previously shown for LSD [21, 22] and MDMA [28, 50].

The present study has strengths. Two well-characterized doses of LSD and psilocybin were used within-subjects and compared with placebo under double-blind conditions in a laboratory setting. We included equal numbers of male and female participants and used internationally established psychometric outcome measures. Plasma LSD and psilocin concentrations were determined up to 24 h for all conditions. Notwithstanding these strengths, the present study also has limitations. The study used a highly controlled setting and included only healthy subjects. Thus, subjects in different environments and patients with psychiatric disorders may respond differently to either LSD or psilocybin.

## Conclusion

We characterized the effects of LSD and psilocybin at two different doses to support dose finding for research and psychedelic-assisted therapy. The 20 mg dose of psilocybin is likely equivalent to the 100 µg dose of LSD base. We found no evidence of qualitative differences in altered states of consciousness that were induced by either LSD or psilocybin, except that the duration of action was shorter for psilocybin.

## Supplementary information

Supplemental Material

Consort Flow Chart
