# Supplementary material for: Direct comparison of the acute effects of lysergic acid diethylamide and psilocybin in a double-blind placebo-controlled study in healthy subjects
Source: Neuropsychopharmacology. 2022 Feb 25;47(6):1180–7. doi: 10.1038/s41386-022-01297-2 (PMC9018810; doi:10.1038/s41386-022-01297-2)
Supplement: Supplementary file 1 — Supplemental Material [file 41386_2022_1297_MOESM1_ESM.docx]

**Supplement**

**Methods**

*Subjective drug effects measurements*

*Visual Analog Scales (VASs)*

Subjective effects were assessed repeatedly using visual analog scales (VASs) [1, 2] 1 h before and 0, 0.25, 0.5, 0.75, 1, 1.5, 2, 2.5, 3, 3.5, 4, 5, 6, 7, 8, 9, 10, 11, 12, 14, 16, and 24 h after drug administration. The VASs included “any drug effect,” “good drug effect,” “bad drug effect,” “drug liking,” “feeling high,” “feeling stimulated,” “fear”, and “ego dissolution,” that were presented as 100-mm horizontal lines (0-100%), marked from “not at all” on the left to “extremely” on the right [1, 3]. Further VASs included “content,” “talkative,” “open,” “concentration,” “speed of thinking,” “perception of time,” “I feel close to others,” “I want to be hugged,” “I want to hug someone,” “I want to be alone”, and “I want to be with others”. “Speed of thinking,” and “perception of time,” were bidirectional and marked with “normal” in the middle at 50 mm and “slowed” on the left (0 mm) and “accelerated” (100 mm) on the right. The VASs “content,” “talkative,” “open,” “concentration,” “I feel close to others,” “I want to be hugged,” “I want to hug someone,” “I want to be alone”, and “I want to be with others” were bidirectional and marked with “normal” in the middle at 50 mm and “not at all” (0 mm) on the left and “extremely” (100 mm) on the right. The primary VAS outcome measures were “any drug effect”, “good drug effect”, “bad drug effect”, and “ego dissolution”. These VAS have been repeatedly used and shown to be sensitive with LSD [1, 2, 4, 5] and were included for the pharmacokinetic-pharmacodynamic modeling as similarly done previously [4, 5]. The VAS can relatively rapidly and easily be completed by the participant during the LSD experience and allow to define the drug effect over time. They are sensitive but relatively simple measures. More complex assessments of the LSD state have to be performed primarily at the end of the session and include entire multi-item questionnaires. The VAS “any drug effect” is an overall effect measure to characterize the overall effect intensity and time course. The VAS “good drug effect” is an overall measure of effects subjectively considered positive and interrelated with other measures such as “drug liking”. The VAS “bad drug effect” is an overall measure of any negative effects and related to “fear”. Typically, “bad drug effects” tend to occur at higher doses or plasma concentrations according to previous PK-PD analyses [4, 5]. The VAS “ego dissolution” was marked with the sentence: “the boundaries between myself and my surroundings seemed to blur”. This is also an item (nr. 71) of the 5D-ASC which has been used as a simple measure of “ego dissolution” previously [6, 7] and can be used repeatedly as single VAS [1, 4].

VASs were assessed each time LSD blood concentrations were measured to allow for pharmacokinetic-pharmacodynamic modeling.

*Adjective Mood Rating Scale (AMRS)*

The Adjective Mood Rating Scale (AMRS) [8] was used 1 h before and 3, 6, 9, 12, and 24 h after drug administration. The AMRS is a validated 60-item Likert mood rating scale mainly use in Europe and consists of subscales including ratings on “well-being”, “anxiety”, “inactivity”, “extraversion”, “introversion”, and “emotional excitation”. It is suitable for repeated measurements of mood states. The short German EWL60S version was used [8]. The completion of the ratings under the effects of psychedelics substances is possible but difficult because it lasts several minutes. The scale was used in paper and pencil version but it may be more suitable to use this measure verbally during states of markedly impaired concentration. The AMRS was included as a secondary supportive measure because it could be considered a better validated measure of mood states and producing more defined ratings than the VAS and to support findings on the VAS (AMRS well-being considered similar to VAS good drug effects; AMRS anxiety considered similar to VAS fear).

*5 Dimensions of Altered States of Consciousness (5D-ASC) scale*

The 5 Dimensions of Altered States of Consciousness (5D-ASC) scale [9, 10] was used as the primary outcome measure and was administered 24 h after drug administration to retrospectively rate peak drug effects. The 5D-ASC scale measures altered states of consciousness and contains 94 items (visual analog scales). The instrument consists of five subscales/dimensions [9] and 11 lower-order scales [10]. The 5D-ASC dimension “Oceanic Boundlessness” (27 items) measures derealization and depersonalization associated with positive emotional states, ranging from heightened mood to euphoric exaltation. The corresponding lower-order scales include “experience of unity,” “spiritual experience,” “blissful state,” “insightfulness,” and “disembodiment.” The dimension “Anxious Ego Dissolution” (21 items) summarizes ego-disintegration and loss of self-control phenomena associated with anxiety. The corresponding lower-order scales include “impaired control of cognition” and “anxiety.” The dimension “Visionary Restructuralization” (18 items) consists of the lower-order scales “complex imagery,” “elementary imagery,” “audio-visual synesthesia,” and “changed meaning of percepts.” Two additional dimensions describe “Auditory Alterations” (15 items) and “Reduction of Vigilance” (12 items). The total 3D-ASC score is the total of the three main dimensions “Oceanic Boundlessness”, “Anxious Ego-Dissolution”, and “Visionary Restructuralization” and can be used as a measure of the overall intensity of the alteration of the mind [7]. The scale is well-validated in German [9] and many other languages and widely used to characterize the subjective effects of various psychedelic drugs. In particular, the scale has been used by most research groups to psychometrically assess LSD effects [1, 2, 11-14]. Furthermore, acute ratings on the 5D-ASC after administration of psilocybin have been used to predict long-term effects of psychedelic treatments in patients [15, 16]. Ratings on the 5D-ASC have been shown to closely correlate with ratings on the Mystical Effects Questionnaire (MEQ, see below) [7] which is primarily used by research groups in the US [16].

*Mystical Effects Questionnaire (MEQ30)*

Mystical experiences were assessed 24 h after drug administration using the 100-item States of Consciousness Questionnaire (SOCQ) [7, 17] that includes the 43-item Mystical Effects Questionnaire (MEQ43) [17], 30-item Mystical Effects Questionnaire (MEQ30) [18], and subscales for “aesthetic experience” and negative “nadir” effects. The published German version was used [7]. The MEQ has been used in numerous experimental and therapeutic trials with psilocybin [16, 17, 19-25]. The MEQ items provide scale scores for each of seven domains of mystical experiences: internal unity, external unity, sacredness, noetic quality (as real as or more real than everyday reality), deeply felt positive mood, transcendence of time and space, and ineffability/paradoxicality (difficulty describing the experience in words). The total of all scale scores was used as an overall measure of the mystical-type experience. We also derived the four scale scores of the newly validated revised 30-item MEQ: mystical, positive mood, transcendence of time and space, and ineffability [18]. A complete mystical experience was defined as scores ≥ 60% on all MEQ30 factors [18]. While we prefer the German 5D-ASC scale, the German version of the MEQ was also included to facilitate comparison of our findings with those from research using the MEQ (mainly US). Additionally, some aspects of the LSD experience may be better captures with this scale. For the scale validation see [18]. For an analysis of the interrelation of the two measures with regards to responses to LSD see [7]. For the German translation of the MEQ30 see online supplement of [7].

**Results**

**
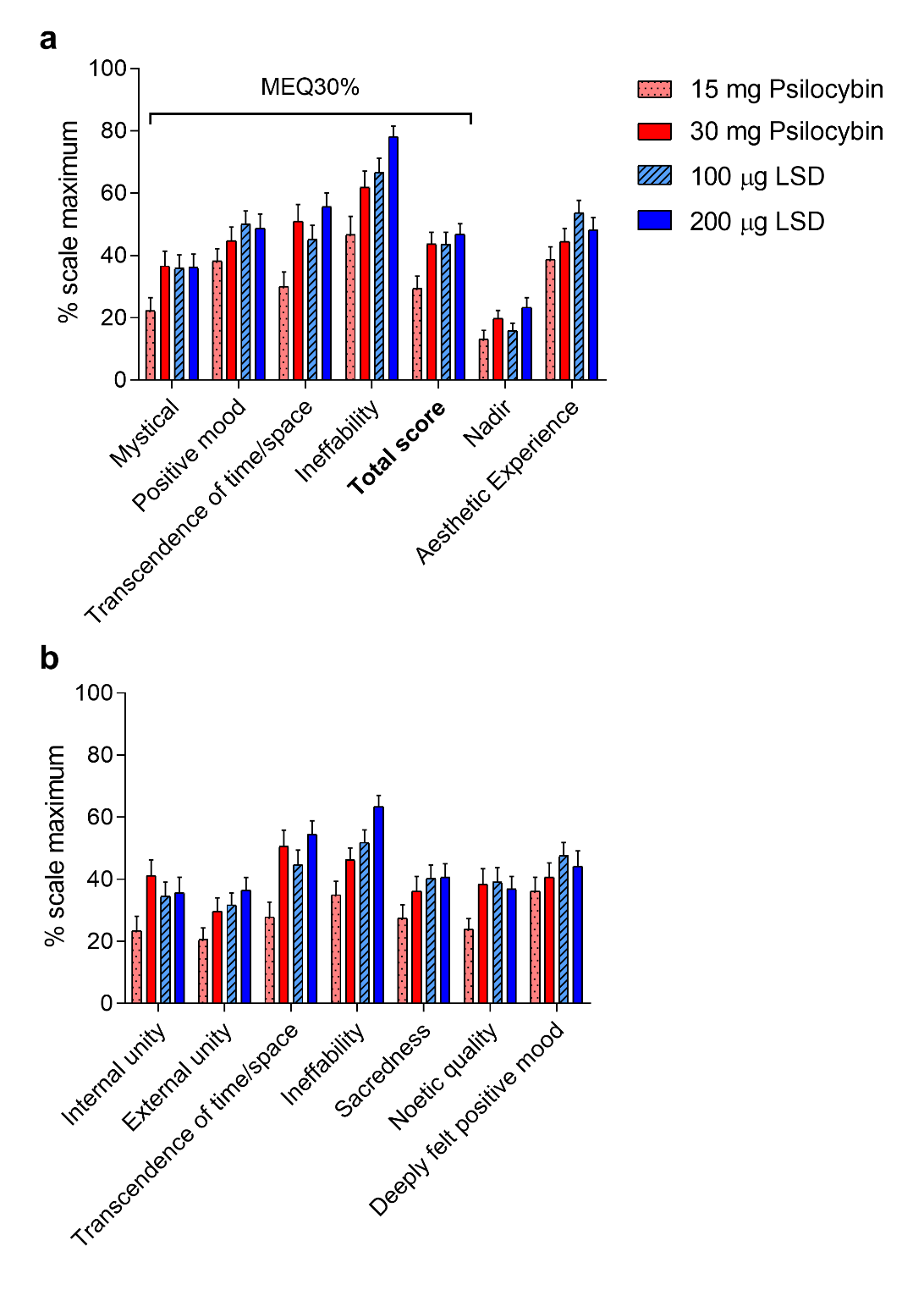
**

**Figure S1.** Acute mystical-type experiences on the Mystical Effects Questionnaire (MEQ). The dose of 30 mg psilocybin induced comparable effects to both 100 and 200 µg of LSD on the MEQ30 (**a**) and the MEQ43 (**b**). The only exceptions were Ineffability on both the MEQ30 and MEQ43 where 200 µg of LSD showed significantly stronger effects than 30 mg of psilocybin. The 15 mg psilocybin dose showed overall clearly weaker effects except for the positive mood subscales both on the MEQ30 and MEQ43. Placebo scores were too low for visualization. The data are expressed as the mean ± SEM percentage of maximally possible scale scores in 28 subjects. Statistics are shown in Supplementary Table S2.


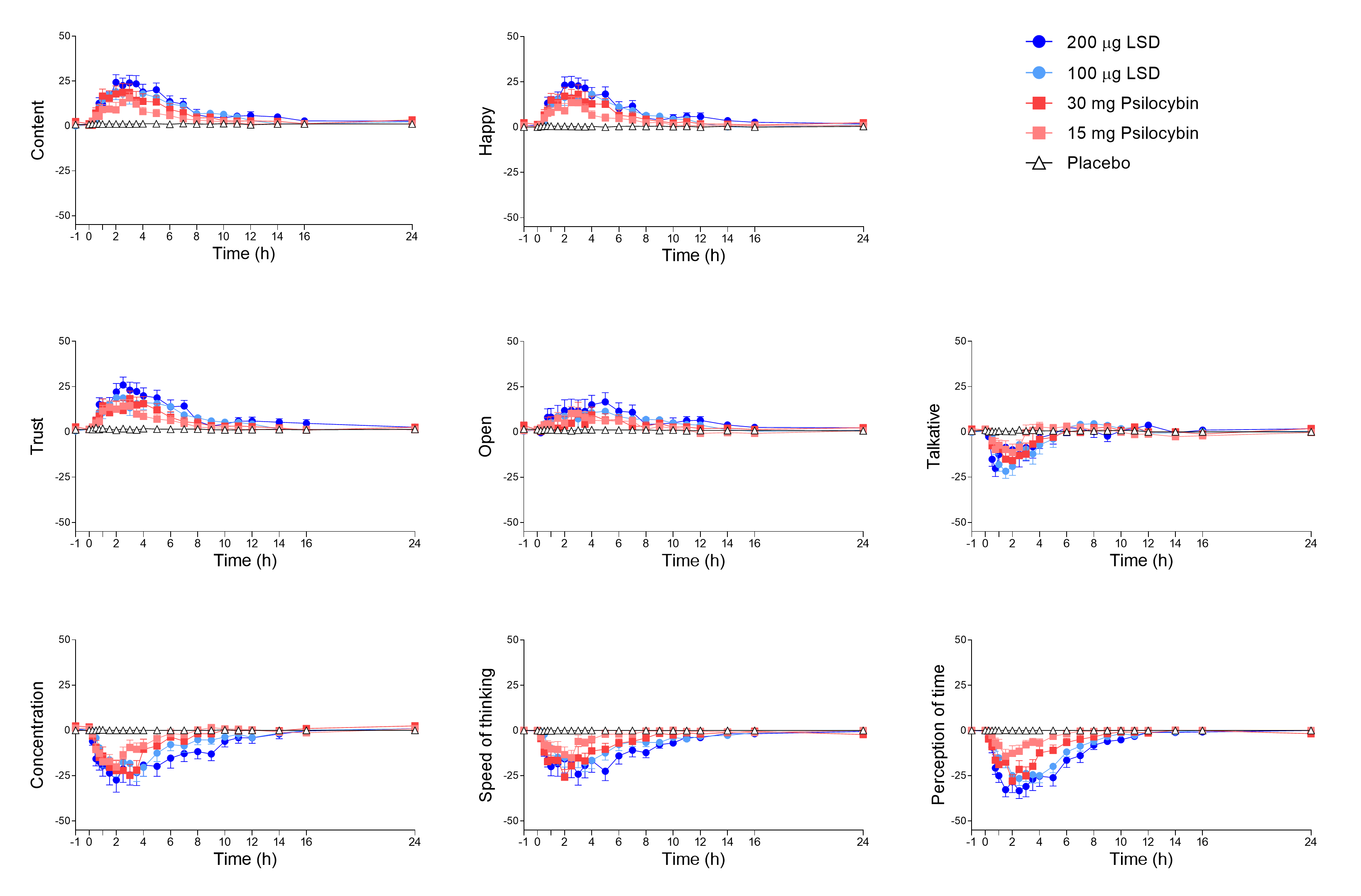

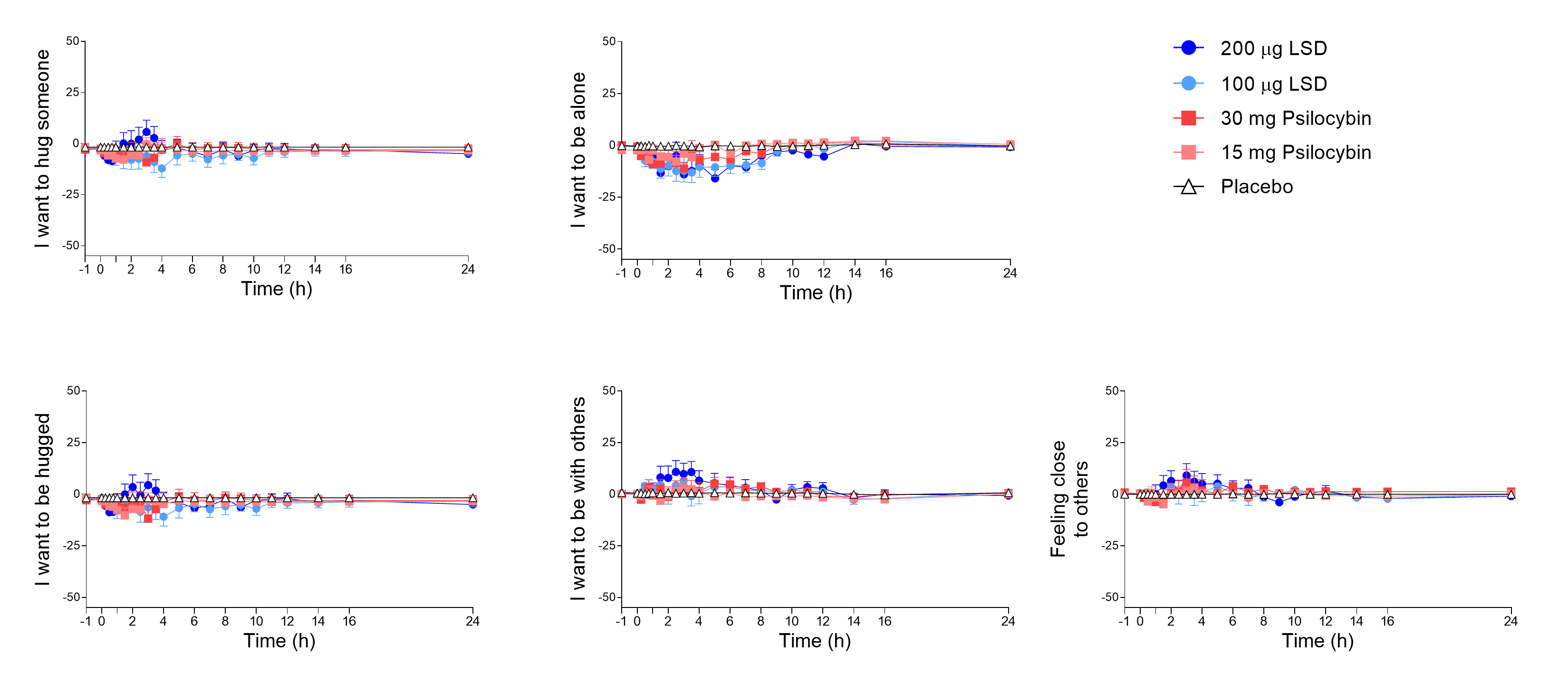


**Figure S2.** Acute subjective effects induced by lysergic acid diethylamide (LSD) or psilocybin over time on the Visual Analog Scale (VAS). LSD (100 or 200 µg), psilocybin (15 or 30 mg), or placebo was administered at t = 0 h. Generally, the LSD doses of 100 µg and 200 µg and the psilocybin dose of 30 mg produced comparable subjective effects on all depicted VASs. Only on the VAS “speed of thinking,” 100 µg and 200 µg LSD showed a significant difference. The high 30 mg psilocybin dose produced comparable maximal subjective effects as the 100 µg or 200 µg LSD dose with no statistical differences on any of the VASs administered. The 30 mg psilocybin dose produced significantly greater peak responses than the 15 mg psilocybin dose on the VASs “speed of thinking” and “perception of time”. The data are expressed as the mean ± SEM percentage of maximally possible scale scores in 28 subjects. The corresponding maximal responses and statistics are shown in Supplementary Table S3.

**
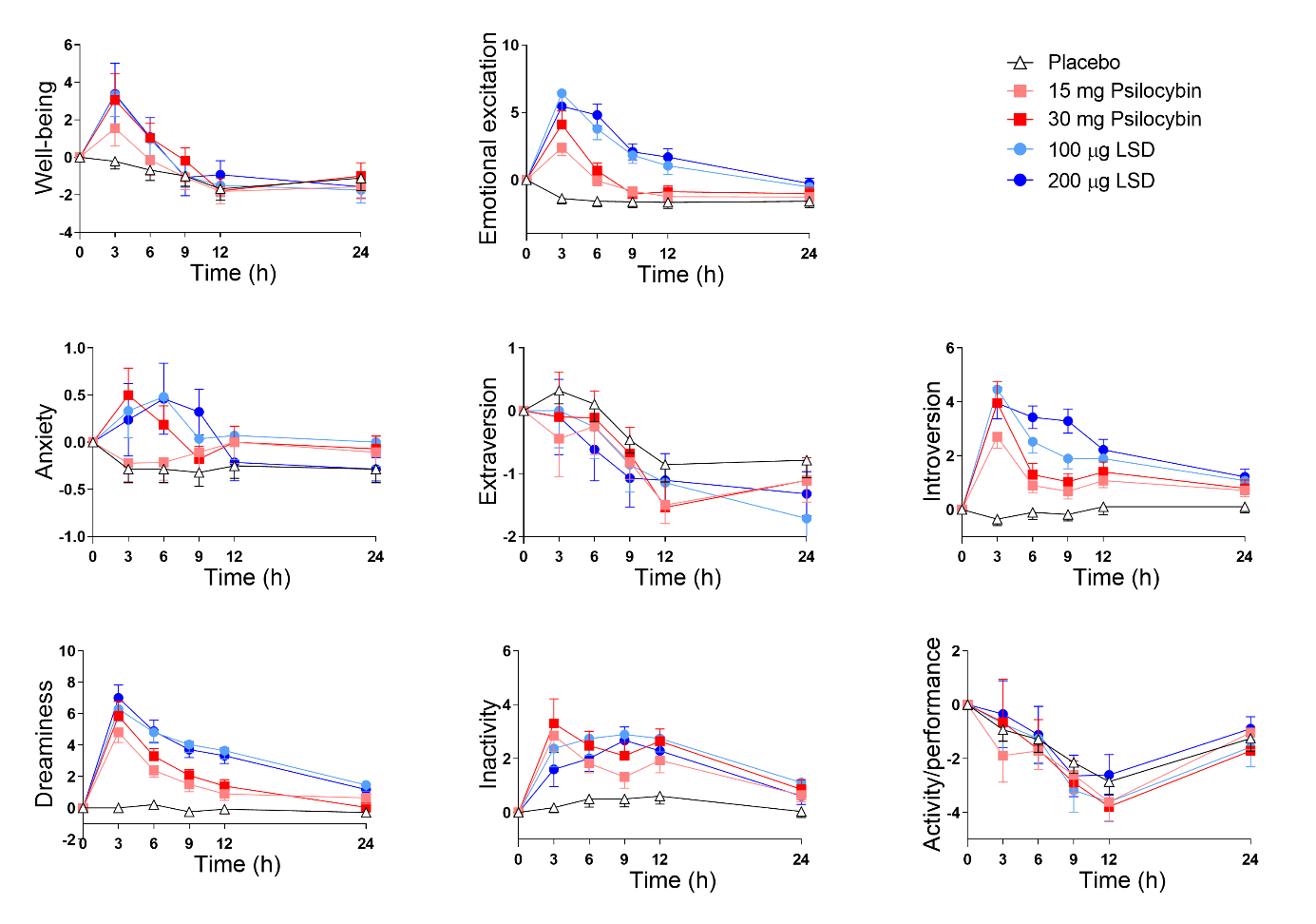
**

**Figure S3**. Subjective effects over time on the Adjective Mood Rating Scale (AMRS). The data are expressed as mean ± SEM changes from baseline. All conditions nominally reduced activity ratings on the AMRS with no difference between placebo and either active drug condition. Both LSD and psilocybin significantly and similarly increased inactivation ratings compared with placebo and at all doses. None of the substances significantly altered well-being ratings although there were nominal increases compared with placebo. Both LSD and psilocybin increased introversion and decrease extraversion ratings at all doses and compared with placebo. Effects of LSD at 100 and 200 µg were greater than those of 15 mg or 30 mg of psilocybin indicating that LSD enhanced emotional excitation more than psilocybin at dose otherwise largely equivalent in terms of other subjective effects. Both doses of LSD and the higher 30 mg psilocybin dose slightly but significantly increased self-rated anxiety. LSD (100 or 200 µg), psilocybin (15 or 30 mg), or placebo was administered at t = 0 h. The corresponding maximal effects and statistics are shown in Supplementary Table S4.


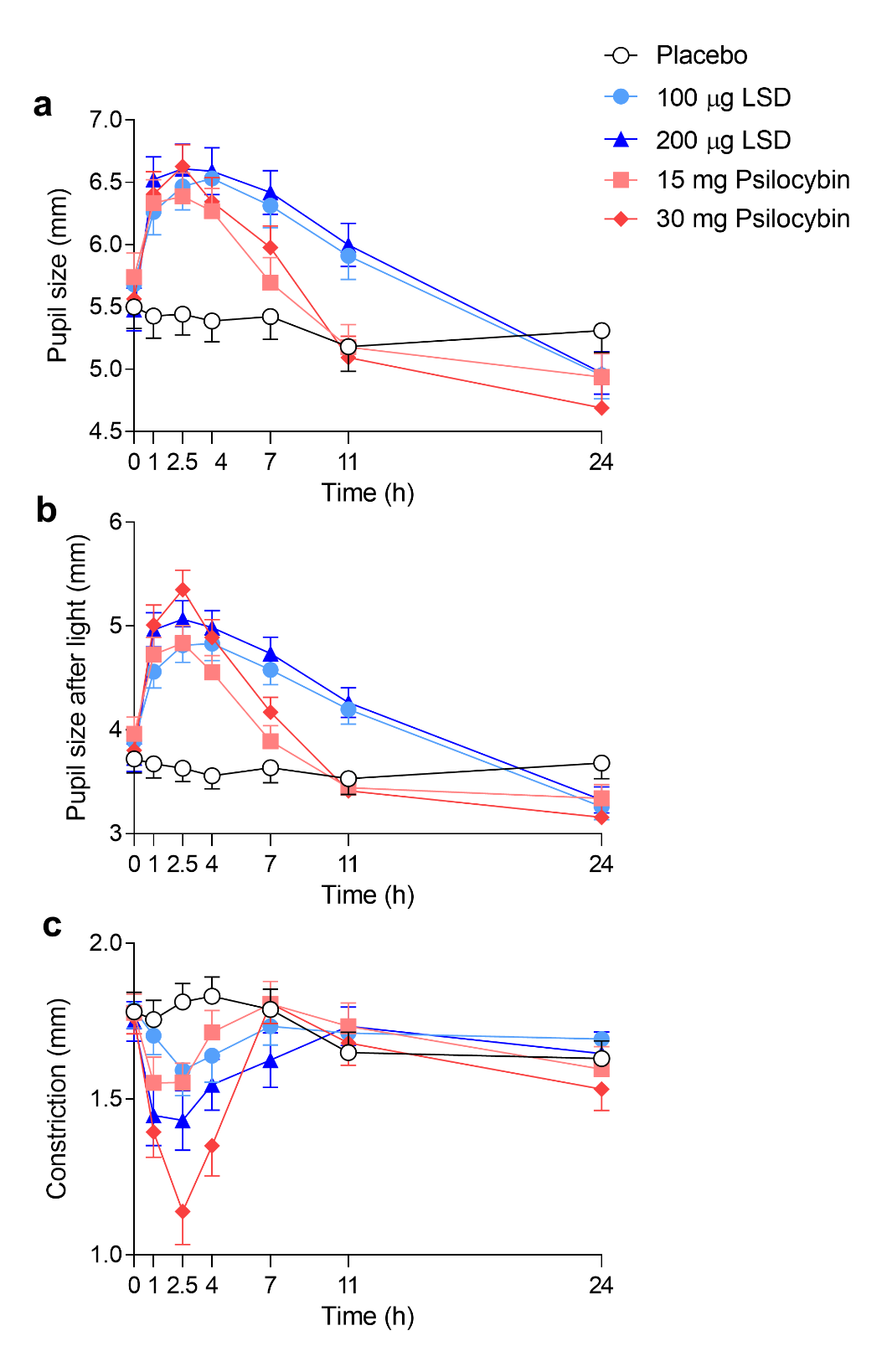


**Figure S4.** Effects of lysergic acid diethylamide (LSD) or psilocybin over time on pupillary function. Both LSD and psilocybin at both doses similarly increased pupil size (**a-b**) at all doses and reduced the reaction to light (**c**). The reduction of the pupillary constriction in response to light was statistically significant more pronounced at the 30 mg psilocybin dose compared with 15 mg of psilocybin, 100 µg LSD and 200 µg LSD. LSD (100 or 200 µg), psilocybin (15 or 30 mg), or placebo was administered at t = 0 h. The data are expressed as the mean ± SEM in 28 subjects. The corresponding maximal effects and statistics are shown in Supplementary Table S5.


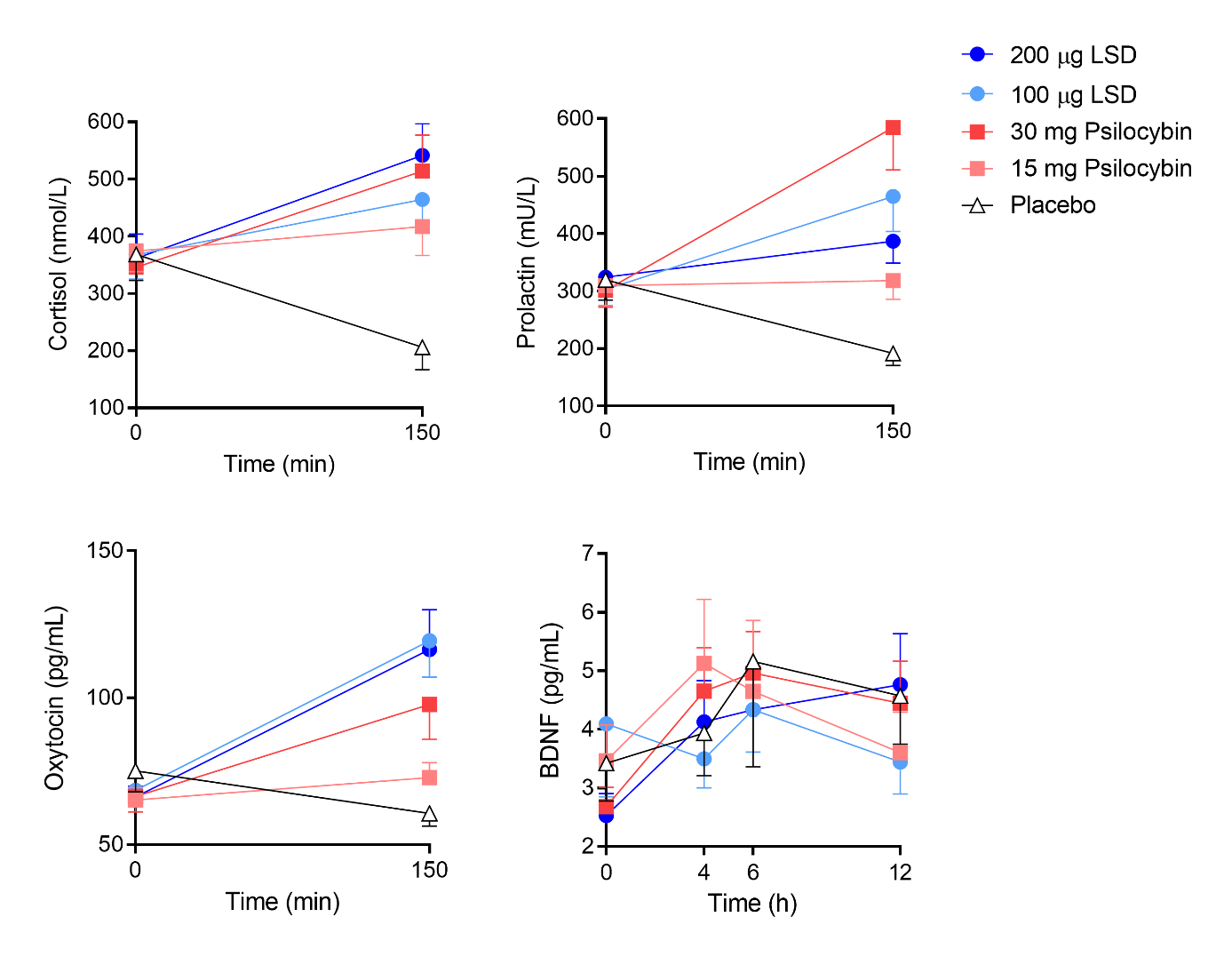


**Figure S5.** Plasma concentrations of cortisol, prolactin, oxytocin, and Brain-Derived Neurotrophic Factor (BDNF). The data are expressed as mean ± SEM. LSD (100 or 200 µg), psilocybin (15 or 30 mg), or placebo was administered at t = 0 h. The corresponding maximal effects and statistics are shown in Supplementary Table S5.


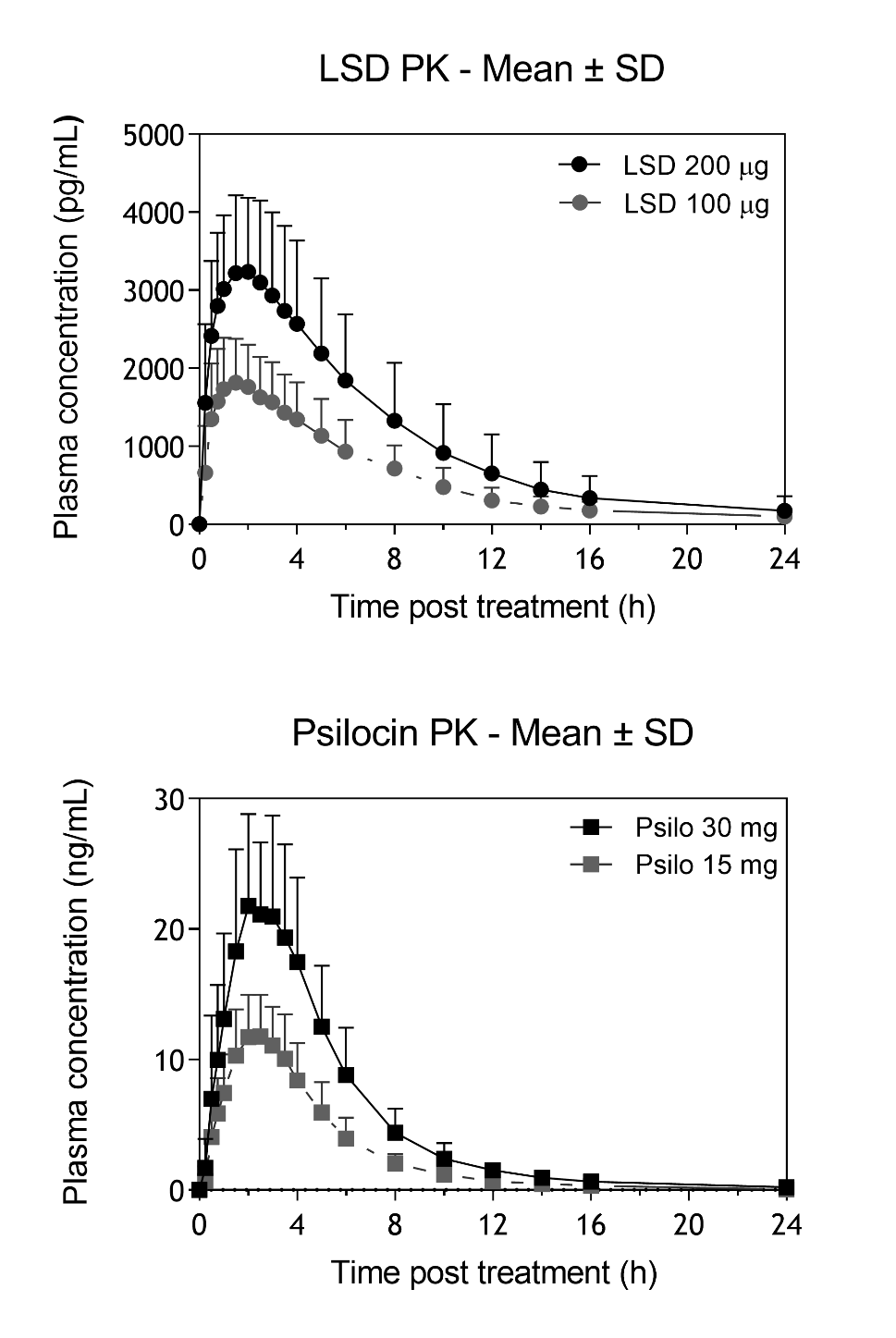
**Figure S6.** Plasma concentrations of LSD and psilocin. The data are expressed as mean ± SEM. LSD (100 or 200 µg), psilocybin (15 or 30 mg), or placebo was administered at t = 0 h. The corresponding pharmacokinetic parameters were determined by non-compartmental analysis and are shown in Supplementary Table S7.

**References**

1. Holze F, Vizeli P, Muller F, Ley L, Duerig R, Varghese N, et al. Distinct acute effects of LSD, MDMA, and D-amphetamine in healthy subjects. Neuropsychopharmacology. 2020;45(3):462-71.

2. Schmid Y, Enzler F, Gasser P, Grouzmann E, Preller KH, Vollenweider FX, et al. Acute effects of lysergic acid diethylamide in healthy subjects. Biol Psychiatry. 2015;78(8):544-53.

3. Hysek CM, Schmid Y, Simmler LD, Domes G, Heinrichs M, Eisenegger C, et al. MDMA enhances emotional empathy and prosocial behavior. Soc Cog Affect Neurosci. 2014;9:1645-52.

4. Holze F, Duthaler U, Vizeli P, Muller F, Borgwardt S, Liechti ME. Pharmacokinetics and subjective effects of a novel oral LSD formulation in healthy subjects. Br J Clin Pharmacol. 2019;85:1474-83.

5. Dolder PC, Schmid Y, Steuer AE, Kraemer T, Rentsch KM, Hammann F, et al. Pharmacokinetics and pharmacodynamics of lysergic acid diethylamide in healthy subjects. Clin Pharmacokinetics. 2017;56:1219-30.

6. Tagliazucchi E, Roseman L, Kaelen M, Orban C, Muthukumaraswamy SD, Murphy K, et al. Increased global functional connectivity correlates with LSD-induced ego dissolution. Curr Biol. 2016;26(8):1043-50.

7. Liechti ME, Dolder PC, Schmid Y. Alterations in conciousness and mystical-type experiences after acute LSD in humans. Psychopharmacology. 2017;234:1499-510.

8. Janke W, Debus G. Die Eigenschaftswörterliste. Göttingen.: Hogrefe; 1978.

9. Dittrich A. The standardized psychometric assessment of altered states of consciousness (ASCs) in humans. Pharmacopsychiatry. 1998;31 (Suppl 2):80-4.

10. Studerus E, Gamma A, Vollenweider FX. Psychometric evaluation of the altered states of consciousness rating scale (OAV). PLoS One. 2010;5(8):e12412.

11. Carhart-Harris RL, Kaelen M, Bolstridge M, Williams TM, Williams LT, Underwood R, et al. The paradoxical psychological effects of lysergic acid diethylamide (LSD). Psychol Med. 2016;46:1379-90.

12. Dolder PC, Schmid Y, Mueller F, Borgwardt S, Liechti ME. LSD acutely impairs fear recognition and enhances emotional empathy and sociality. Neuropsychopharmacology. 2016;41:2638-46.

13. Bershad AK, Schepers ST, Bremmer MP, Lee R, de Wit H. Acute subjective and behavioral effects of microdoses of lysergic acid diethylamide in healthy human volunteers. Biol Psychiatry. 2019;86(10):792-800.

14. Preller KH, Herdener M, Pokorny T, Planzer A, Kraehenmann R, Stämpfli P, et al. The fabric of meaning and subjective effects in LSD-induced states depend on serotonin 2A receptor activation. Curr Biol. 2017;27:451-57.

15. Roseman L, Nutt DJ, Carhart-Harris RL. Quality of acute psychedelic experience predicts therapeutic efficacy of psilocybin for treatment-resistant depression. Front Pharmacol. 2017;8:974.

16. Griffiths RR, Johnson MW, Carducci MA, Umbricht A, Richards WA, Richards BD, et al. Psilocybin produces substantial and sustained decreases in depression and anxiety in patients with life-threatening cancer: a randomized double-blind trial. J Psychopharmacol. 2016;30(12):1181-97.

17. Griffiths RR, Richards WA, McCann U, Jesse R. Psilocybin can occasion mystical-type experiences having substantial and sustained personal meaning and spiritual significance. Psychopharmacology. 2006;187(3):268-83; discussion 84-92.

18. Barrett FS, Johnson MW, Griffiths RR. Validation of the revised Mystical Experience Questionnaire in experimental sessions with psilocybin. J Psychopharmacol. 2015;29(11):1182-90.

19. MacLean KA, Johnson MW, Griffiths RR. Mystical experiences occasioned by the hallucinogen psilocybin lead to increases in the personality domain of openness. J Psychopharmacol. 2011;25(11):1453-61.

20. Griffiths RR, Johnson MW, Richards WA, Richards BD, McCann U, Jesse R. Psilocybin occasioned mystical-type experiences: immediate and persisting dose-related effects. Psychopharmacology. 2011;218(4):649-65.

21. Griffiths R, Richards W, Johnson M, McCann U, Jesse R. Mystical-type experiences occasioned by psilocybin mediate the attribution of personal meaning and spiritual significance 14 months later. J Psychopharmacol. 2008;22(6):621-32.

22. Garcia-Romeu A, Griffiths RR, Johnson MW. Psilocybin-occasioned mystical experiences in the treatment of tobacco addiction. Curr Drug Abuse Rev. 2015;7(3):157-64.

23. Garcia-Romeu A, Davis AK, Erowid F, Erowid E, Griffiths RR, Johnson MW. Cessation and reduction in alcohol consumption and misuse after psychedelic use. J Psychopharmacol. 2019:269881119845793.

24. Griffiths RR, Johnson MW, Richards WA, Richards BD, Jesse R, MacLean KA, et al. Psilocybin-occasioned mystical-type experience in combination with meditation and other spiritual practices produces enduring positive changes in psychological functioning and in trait measures of prosocial attitudes and behaviors. J Psychopharmacol. 2018;32:49-69.

25. Ross S, Bossis A, Guss J, Agin-Liebes G, Malone T, Cohen B, et al. Rapid and sustained symptom reduction following psilocybin treatment for anxiety and depression in patients with life-threatening cancer: a randomized controlled trial. J Psychopharmacol. 2016;30(12):1165-80.
