# Supplementary material for: Direct comparison of the acute effects of lysergic acid diethylamide and psilocybin in a double-blind placebo-controlled study in healthy subjects
Source: Neuropsychopharmacology. 2022 Feb 25;47(6):1180–7. doi: 10.1038/s41386-022-01297-2 (PMC9018810; doi:10.1038/s41386-022-01297-2)
Supplement: Supplementary file 2 — Consort Flow Chart [file 41386_2022_1297_MOESM2_ESM.pptx]

## Slide 1
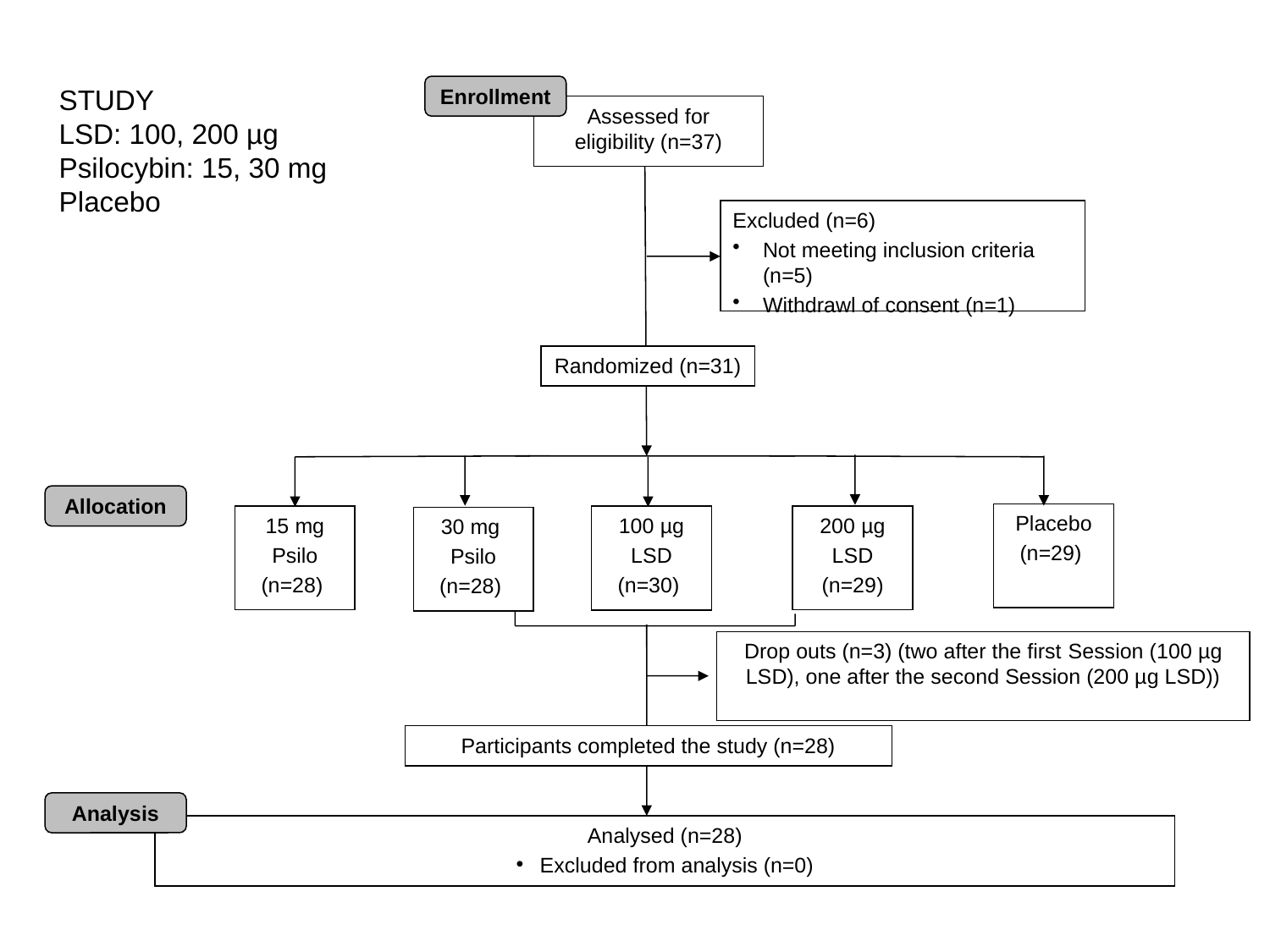

STUDY
LSD: 100, 200 µg
Psilocybin: 15, 30 mg
Placebo
Enrollment
Assessed for eligibility (n=37)
Excluded (n=6)
Not meeting inclusion criteria (n=5)
Withdrawl of consent (n=1)
Randomized (n=31)
Allocation
Placebo
(n=29)
15 mg
Psilo
(n=28)
200 µg
LSD
(n=29)
100 µg
LSD
(n=30)
30 mg
Psilo
(n=28)
Drop outs (n=3) (two after the first Session (100 µg LSD), one after the second Session (200 µg LSD))
Participants completed the study (n=28)
Analysis
Analysed (n=28)
Excluded from analysis (n=0)
